# Highly conserved binding region of ACE2 as a receptor for SARS-CoV-2 between humans and mammals

**DOI:** 10.1080/01652176.2020.1823522

**Published:** 2020-09-29

**Authors:** Takuma Hayashi, Kaoru Abiko, Masaki Mandai, Nobuo Yaegashi, Ikuo Konishi

**Affiliations:** a National Hospital Organization Kyoto Medical Center, Kyoto, Japan; b START, Japan Science and Technology Agency (JST), Tokyo, Japan; c Department of Obstetrics and Gynecology, Kyoto University School of Medicine, Kyoto, Japan; d Department of Obstetrics and Gynecology, Tohoku University School of Medicine, Miyagi, Japan; e Immediate Past President of Asian Society of Gynecologic Oncology, Tokyo, Japan

**Keywords:** SARS-CoV-2, COVID-19, ACE2, binding region, mink

## Abstract

Several cases of severe acute respiratory syndrome coronavirus 2 (SARS-CoV-2) infection transmitted from human owners to their dogs have recently been reported. The first ever case of SARS-CoV-2 transmission from a human owner to a domestic cat was confirmed on March 27, 2020. A tiger from a zoo in New York, USA, was also reportedly infected with SARS-CoV-2. It is believed that SARS-CoV-2 was transmitted to tigers from their caretakers, who were previously infected with this virus. On May 25, 2020, the Dutch Minister of Agriculture, Nature and Food Quality reported that two employees were infected with SARS-CoV-2 transmitted from minks. These reports have influenced us to perform a comparative analysis among angiotensin-converting enzyme 2 (ACE2) homologous proteins for verifying the conservation of specific protein regions. One of the most conserved peptides is represented by the peptide “353-KGDFR-357 (*H. sapiens* ACE2 residue numbering), which is located on the surface of the ACE2 molecule and participates in the binding of SARS-CoV-2 spike receptor binding domain (RBD). Multiple sequence alignments of the ACE2 proteins by ClustalW, whereas the three-dimensional structure of its binding region for the spike glycoprotein of SARS-CoV-2 was assessed by means of Spanner, a structural homology modeling pipeline method. In addition, evolutionary phylogenetic tree analysis by ETE3 was used. ACE2 works as a receptor for the SARS-CoV-2 spike glycoprotein between humans, dogs, cats, tigers, minks, and other animals, except for snakes. The three-dimensional structure of the KGDFR hosting protein region involved in direct interactions with SARS-CoV-2 spike RBD of the mink ACE2 appears to form a loop structurally related to the human ACE2 corresponding protein loop, despite of the reduced available protein length (401 residues of the mink ACE2 available sequence vs 805 residues of the human ACE2). The multiple sequence alignments of the ACE2 proteins shows high homology and complete conservation of the five amino acid residues: 353-KGDFR-357 with humans, dogs, cats, tigers, minks, and other animals, except for snakes. Where the information revealed from our examinations can support precision vaccine design and the discovery of antiviral therapeutics, which will accelerate the development of medical countermeasures, the World Health Organization recently reported on the possible risks of reciprocal infections regarding SARS-CoV-2 transmission from animals to humans.

## 1. Introduction

A novel human coronavirus that has now been termed severe acute respiratory syndrome coronavirus 2 (SARS-CoV-2) (formerly called 2019-nCoV) first emerged in Wuhan, China, in late 2019, and has since then led to a worldwide pandemic (WHO 2020). The genome of SARS-CoV-2 shares approximately 80% of identity with that of SARS-CoV and is approximately 96% identical to the bat coronavirus BatCoV RaTG13 (Zhou et al. 2020). In the case of SARS-CoV, the spike glycoprotein on the virion surface mediates receptor recognition and membrane fusion (Gallagher and Buchmeier 2001; Simmons et al. 2013; Yan et al. 2020). During viral infection, the trimeric spike glycoprotein is cleaved into S1 and S2 subunits, where S1 contains the receptor binding domain (RBD), which directly binds to the region located in the peptidase domain of the angiotensin-converting enzyme 2 (ACE2), where S2 subunits are responsible for membrane fusion (Lu et al. 2020; Mercurio et al. 2020; Yan et al. 2020). S1 subunits are released in transition to the post-fusion conformation (Simmons et al. 2013; Lu et al. 2020; Mercurio et al. 2020; Yan et al. 2020).

Several cases of SARS-CoV-2 infection transmitted from human owners to their dogs have been reported in Hong Kong (Sit et al. 2020). Cases of the new coronavirus (SARS-CoV-2) infection transmitted from human owners to their domestic cats were also reported on March 27, 2020, in Belgium. This is the first instance in history that cases of SARS-CoV-2 transmission from human owners to their domestic cats have been confirmed (AP News 2020). A tiger kept at a zoo in New York, USA, was also reportedly infected with SARS-CoV-2. It is believed that SARS-CoV-2 has been transmitted to tigers from caretakers who were previously infected with this virus. It has been confirmed that minks bred on four farms near Eindhoven in Northern Brabant, Southern Netherlands, were infected with SARS-CoV-2, although the transmission of human coronavirus to pets and/or other animals is rare. At the same time, the World Health Organization (WHO) has reported that the dangers of transmitting SARS-CoV-2 from animals or pets to humans have not yet been proven. Then, in April 2020, the four above-mentioned farms were closed. On April 25, 2020, the Dutch Minister of Agriculture, Nature and Food Quality reported that two employees were infected with SARS-CoV-2 transmitted from minks at the farms. On April 26, 2020, the WHO reported the first case of coronavirus infection disease-2019 (COVID-19) transmitted from animals to humans (Dutch mink workers… 2020).

## 2. Methods

Multiple sequence alignments of the ACE2 proteins were performed by ClustalW, whereas the three-dimensional structure of its binding region for the spike glycoprotein of SARS-CoV-2 was assessed by means of Spanner, a structural homology modeling pipeline method. In addition, evolutionary phylogenetic tree analysis by ETE3 was used.

## 3. Results

**Figure 1.**
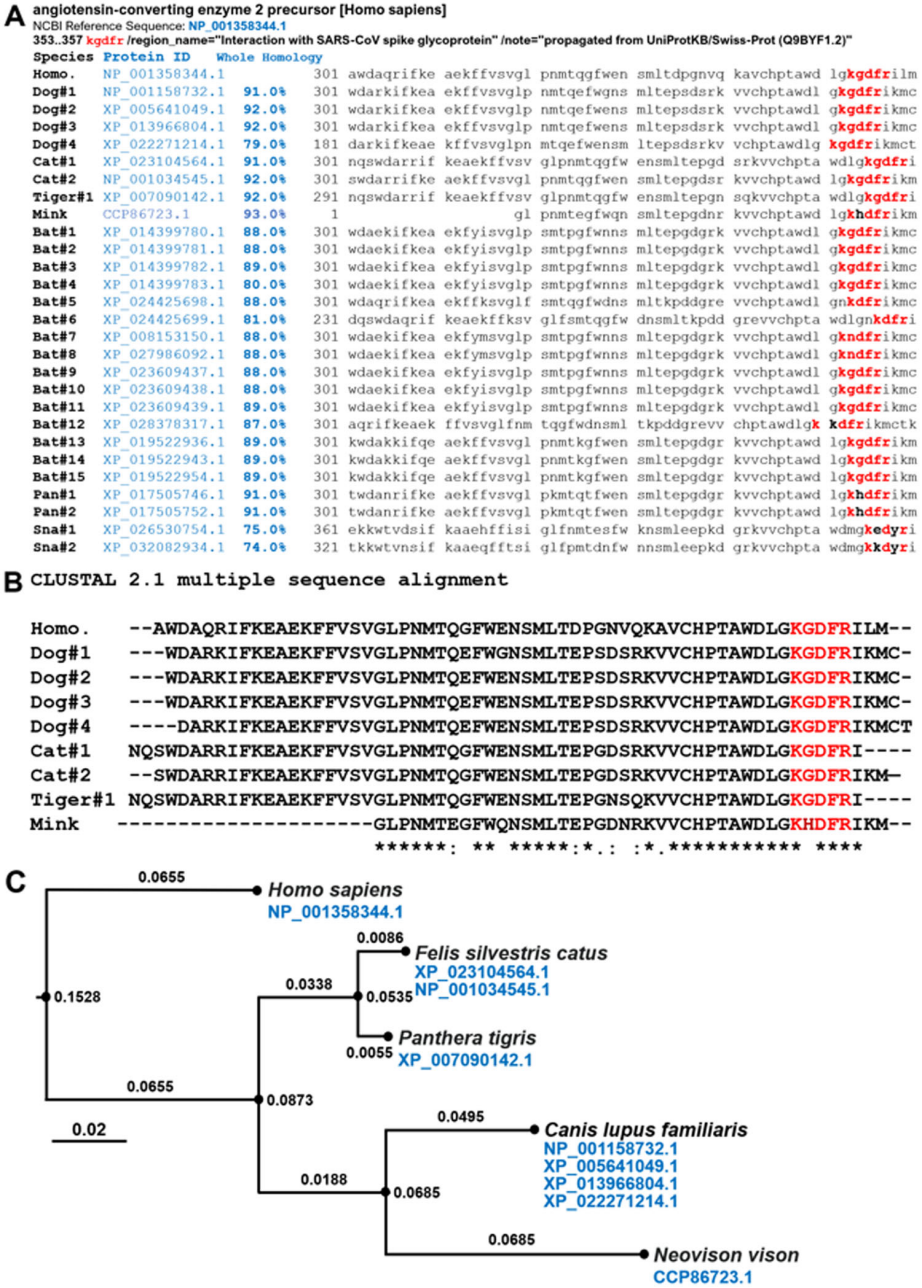
Amino acid sequence alignment of angiotensin-converting enzyme 2 (ACE2) binding region working as a receptor for SARS-CoV-2 spike glycoprotein and its phylogenetic tree. (A) The whole molecule homology and homologous binding region of angiotensin-converting enzyme 2 (ACE2) between humans and other animal species including dogs, cats, tigers, minks, bats, pangolins, and snakes are indicated in the Supplementary Materials section. The key five amino acid residues 353-KGDFR-357 involved in the interaction with human SARS-CoV-2 spike glycoprotein are marked in red. Detailed information regarding the protein accession numbers of ACE2 in other animal species can be found in the Supplementary Materials section. (B) The multiple sequence alignments of the ACE2 proteins by ClustalW revealed the complete conservation of the five amino acid residues: 353-KGDFR-357 between humans, dogs, cats, tigers, and minks. (C) The evolutionary phylogenetic tree analysis by ETE3 shows that mink ACE2 (Mustelidae family) sequence locates close to canis ACE2 sequences, SARS-CoV-2 is considered to be transmitted between humans and minks.

**Figure 2.**
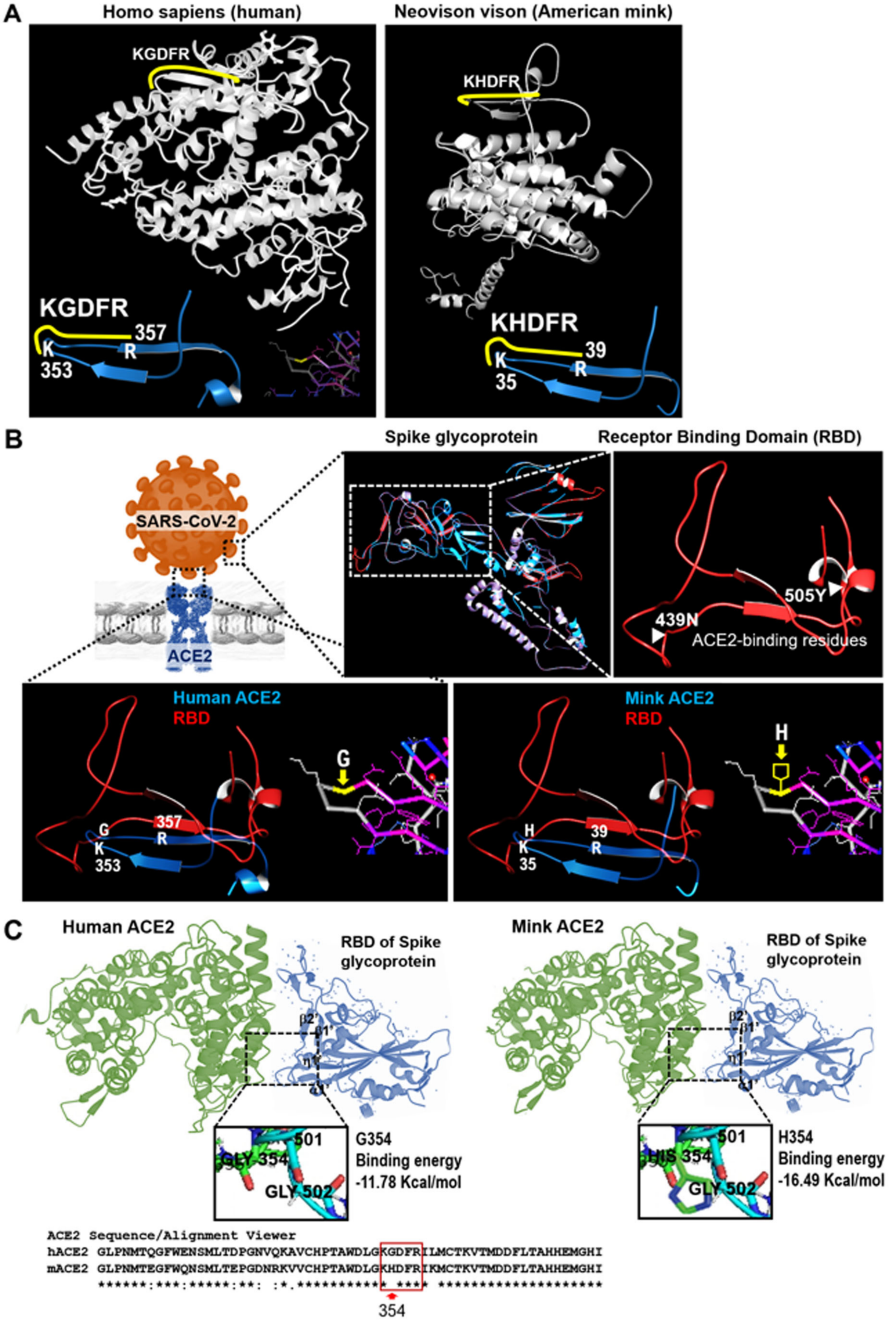
The complex structure of SARS-CoV-2-CTD bound to human or mink ACE2. (A) Three-dimensional structures of the binding region for mink and human ACE2s with the spike glycoprotein of SARS-CoV-2 using Spanner, a structural homology modeling pipeline method (Supplementary Materials). The three-dimensional structure of the binding region containing five amino acids of mink ACE2 indicated by the yellow line involved in binding interactions with the spike glycoprotein of SARS-CoV-2 is conserved in comparison with the structure of human ACE2. The ribbon diagram highlights the native human ACE2 with the secondary structure and the two subdomains (I and II) that form the two sides of the active site cleft. The two subdomains of human ACE2 are defined as follows: the N terminus- and zinc-containing subdomain I, composed of residues 290–397, and 417–430, and the C terminus-containing subdomain II, composed of residues 103–289, 398–416, and 431–615 (Towler et al. 2004). The protein ID numbers NP_001358344.1 for human ACE2 and CCP86723.1 for mink ACE2 are used to analyze the three-dimensional structures for mink and human ACE2 molecules. Mink ACE2 (protein ID CCP86723.1) is 471 aa long instead of the 805 aa of the human sequence. We prepared the 3D homology model of the available mink partial sequence by using the human ACE2 as a structure template obtaining the 3D model of the mink 471 aa long sequence. Details of the amino acid sequence of mink and human ACE2s are shown in the Supplementary Materials section. (B) Three-dimensional structures of the spike glycoprotein of SARS-CoV-2 in complex with ACE2 using Spanner analysis (Supplementary Materials). The three-dimensional structures of receptor binding domain (RBD) of the spike glycoprotein of SARS-CoV-2 are displayed by the red line at the top right panel in B. The predictions of the complex structure of the RBD (red line) with the protein region including the five amino acids of human and mink ACE2s (blue line) are shown in the bottom part of panel B. The pictures of the protein region including the five amino acids of human and mink ACE2s obtained from the Cn3D macromolecular structure viewer are shown in the squared boxes. Details of the amino acid sequence of mink, and human ACE2s are shown in the Supplementary Materials section. (C) A cartoon representation of the complex structure is analyzed using the LigPlot + program (v.1.4.5) and MOE project DB (MOLSIS Inc., Tokyo, Japan). The core and external subdomains in RBD in the spike glycoprotein of SARS-CoV-2 are colored in cyan. hACE2 subdomains I and II are green, respectively. Key contact sites are marked with the left and right boxes in three-dimensional structures and are further delineated for interaction details, respectively. The homology modeling of human (left) and mink (right) with SARS-CoV-2 RBD with G354 (left) or H354 (right) residue are reported. Protein buried surface areas are analyzed using the PDBePISA tool and MOE project DB (MOLSIS Inc., Tokyo, Japan).

**Figure 3.**
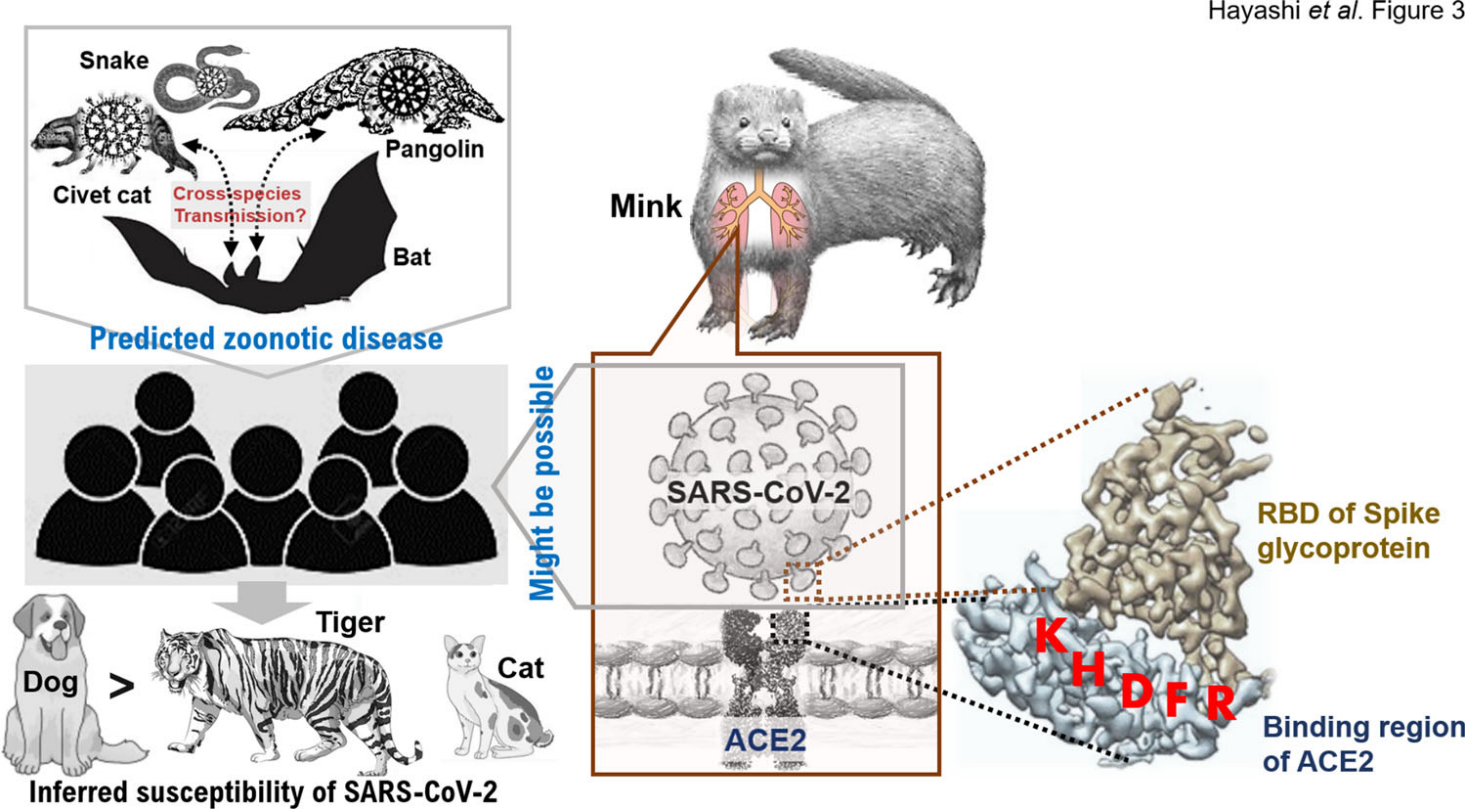
SARS-CoV-2 infection from mink to human in zoonotic transmission. Currently, the exact route of zoonotic transmission of SARS-CoV-2 into the human population remains unknown. Structural analysis suggests that multiple introductions of SARS-CoV-2 into the human population have occurred and both zoonotic transmission events and human-to-human transmission drive the current COVID-19 outbreak. Previous research papers have reported that SARS-CoV-2 had a higher rate of infection in canines compared to the rate of infection in felines (Patterson et al. 2020). SARS-CoV-2 may be more likely to infect canines than felines.

Recent reports have highlighted that cats are experimentally susceptible to airborne infections (Halfmann et al. 2020; Shi et al. 2020). The multiple sequence alignments of the sampled ACE2 proteins revealed high homology and high conservation of the five amino acid residues: 353-KGDFR-357 with specific reference to humans, dogs, cats, tigers, minks, and other animals, except for snakes (Figure 1(A) and Supplementary Materials). Therefore, we performed a comparative analysis among ACE2 homologous proteins for verifying the conservation of specific protein regions at structural level. ACE2 works as a receptor for the SARS-CoV-2 spike glycoprotein between humans, dogs, cats, tigers, minks, and other mammals. The multiple sequence alignments of the ACE2 proteins by ClustalW revealed the complete conservation of the five amino acid residues: 353-KGDFR-357 between humans, dogs, cats, tigers, and minks (Figure 1(B)). To date, SARS-CoV-2 from human owners has been reported to infect their dogs (Sit et al. 2020). According to the phylogenetic tree, the mink ACE2 sequence cluster is phylogenetically very close to dog sequence cluster. We therefore suppose that SARS-CoV-2 can infect minks with the same likelihood SARS-CoV-2 can infect dogs (Figure 1(C)). We analyzed the three-dimensional structure of the binding region in mink and human ACE2s (hACE2) for the spike glycoprotein of SARS-CoV-2 using Spanner, a structural homology modeling pipeline method (Supplementary Materials). The three-dimensional structure of the binding region containing the investigated five amino acids of mink ACE2 involved in binding interactions with the spike glycoprotein of SARS-CoV-2 was conserved in comparison with the structure of hACE2 (Figure 2(A)). The complex structure data show that the RBD of SARS-CoV-2 utilizes its external subdomain to recognize subdomain I in the hACE2 (Figure 2(C)), with the analysis revealing that the three-dimensional structure of the binding region of mink ACE2 complements perfectly the structure of the RBD of the spike glycoprotein of SARS-CoV-2 (Figure 2(B)). Notably, the bulged loops in the RBD of SARS-CoV-2, that is, α1′/β1′ loop and β2’/η1′ loop, properly position several residues (G446, Y449, G496, Q498, T500, and G502) in close proximity with hACE2 amino acids D38, Y41, Q42, K353, G354, and D355 or mink ACE2 amino acid H354, forming a concentration of H bonds (Figure 2(C)). Structural remodeling also suggested that the G354H substitution in five key amino acid residues in the surface motif of mink ACE2 increased the binding affinity of the RBD of SARS-CoV-2 (Figure 2(C)). As the binding free energy decreases, the affinity between ligand and receptor increases. Overall, the virus–receptor engagement is dominated by polar contacts mediated by the hydrophilic residues. In support of this hypothesis, a single G354H substitution was sufficient to strongly conserve these interactions (Figure 2(C)). Our research results suggest that SARS-CoV-2 zoonotic disease may spread transmitting among different animal species worldwide (Figure 3).

Some strains of CoV are zoonotic, meaning they can be transmitted between animals and humans, but several strains are not zoonotic. In humans, CoV can cause illness ranging from the common cold to more severe diseases such as Middle East respiratory syndrome (caused by MERS-CoV), and severe acute respiratory syndrome (caused by SARS-CoV). Detailed investigations have demonstrated that SARS-CoV and MERS-CoV were transmitted from civets to humans and from dromedary camels to humans, respectively (Guan et al. 2003; Azhar et al. 2014) (Figure 3). Where current evidence suggests that SARS-CoV-2 emerged from an animal source, the current pandemic is being sustained through human-to-human transmission of SARS-CoV-2. While there is to date insufficient scientific evidence to identify the source of SARS-CoV-2 or explain the original route of transmission to humans, genetic sequence data reveal that SARS-CoV-2 is a close relative of other CoV found circulating in Rhinolophus bat or pangolin populations (Cyranoski 2020; Zhou et al. 2020).

## 4. Discussion

Priorities for research to investigate the animal source were discussed by the Office International des Epizooties (OIE) *ad hoc* Group on COVID-19 at the Human–Animal Interface and were presented at the WHO Global Research and Innovation Forum (February 11–12, 2020) by the president of the OIE Wildlife Working Group (https://www.oie.int/scientific-expertise/specific-information-and-recommendations/questions-and-answers-on-2019novel-coronavirus/). While, in the field setting, cats have shown clinical signs of disease including respiratory and gastrointestinal signs (AP News 2020), cats (domestic and large cats), mink, and dogs have tested positive for SARS-CoV-2 in these field settings, following contact with humans known or suspected to be infected with SARS-CoV-2 (AP News 2020; Daly N 2020; Enserink 2020; Sit et al. 2020; https://www.oie.int/scientific-expertise/specific-information-and-recommendations/questions-and-answers-on-2019novel-coronavirus/) (Figure 3). While there is no evidence that companion animals are playing an epidemiological role in the spread of human infections with SARS-CoV-2, SARS-CoV-2 infection in farmed minks has been characterized by respiratory disease and an increased mortality rate (Enserink 2020). Reports from infected mink farms therefore suggest that, in these environments, there is the possibility for transmission of SARS-CoV-2 from minks to humans (Enserink 2020; https://www.oie.int/scientific-expertise/specific-information-and-recommendations/questions-and-answers-on-2019novel-coronavirus/). Studies are underway to better understand the susceptibility of different animal species to SARS-CoV-2 and assess infection dynamics in susceptible animal species.

Of note, the complex of spike glycoprotein of SARS-CoV-2 and ACE2 translocate into endosomes in cytosol of host cell. Therefore, this binding might be without consequences for the function of the ACE2 receptor.

## 5. Conclusions

Alongside recent studies, our findings may provide significant insights into animal models for SARS-CoV-2 and animal management for COVID-19 control. The WHO considers zoonotic infections to be special cases. Where the information provided from our examinations will support precision vaccine design and the discovery of antiviral therapeutics, which will, in turn, accelerate the development of medical countermeasures, the WHO has called for individuals to also avoid cross transmission of SARS-CoV-2 between humans, their pets, and other animals.
